# Computational approaches for evaluating morphological changes in the corneal stroma associated with decellularization

**DOI:** 10.3389/fbioe.2023.1105377

**Published:** 2023-05-26

**Authors:** Igor V. Pantic, Jelena Cumic, Svetlana Valjarevic, Adeeba Shakeel, Xinyu Wang, Hema Vurivi, Sayel Daoud, Vincent Chan, Georg A. Petroianu, Meklit G. Shibru, Zehara M. Ali, Dejan Nesic, Ahmed E. Salih, Haider Butt, Peter R. Corridon

**Affiliations:** ^1^ Department of Medical Physiology, Faculty of Medicine, Visegradska 26/II, University of Belgrade, Belgrade, Serbia; ^2^ University of Haifa, Haifa, Israel; ^3^ Department of Physiology and Cell Biology, Faculty of Health Sciences, Ben-Gurion University of the Negev, Be’er Sheva, Israel; ^4^ Department of Pharmacology, College of Medicine and Health Sciences, Khalifa University of Science and Technology, Abu Dhabi, United Arab Emirates; ^5^ Faculty of Medicine, University of Belgrade, University Clinical Center of Serbia, Belgrade, Serbia; ^6^ Faculty of Medicine, Clinical Hospital Center Zemun, University of Belgrade, Belgrade, Serbia; ^7^ Department of Immunology and Physiology, College of Medicine and Health Sciences, Khalifa University of Science and Technology, Abu Dhabi, United Arab Emirates; ^8^ Biomedical Engineering, Healthcare Engineering Innovation Center, Khalifa University of Science and Technology, Abu Dhabi, United Arab Emirates; ^9^ Center for Biotechnology, Khalifa University of Science and Technology, Abu Dhabi, United Arab Emirates; ^10^ Anatomical Pathology Laboratory, Cleveland Clinic Abu Dhabi, Abu Dhabi, United Arab Emirates; ^11^ Department of Mechanical Engineering, College of Medicine and Health Sciences, Khalifa University of Science and Technology, Abu Dhabi, United Arab Emirates

**Keywords:** cornea, corneal stroma, decellualrization, GLCM (gray level co-event matrix), keratocytes

## Abstract

Decellularized corneas offer a promising and sustainable source of replacement grafts, mimicking native tissue and reducing the risk of immune rejection post-transplantation. Despite great success in achieving acellular scaffolds, little consensus exists regarding the quality of the decellularized extracellular matrix. Metrics used to evaluate extracellular matrix performance are study-specific, subjective, and semi-quantitative. Thus, this work focused on developing a computational method to examine the effectiveness of corneal decellularization. We combined conventional semi-quantitative histological assessments and automated scaffold evaluations based on textual image analyses to assess decellularization efficiency. Our study highlights that it is possible to develop contemporary machine learning (ML) models based on random forests and support vector machine algorithms, which can identify regions of interest in acellularized corneal stromal tissue with relatively high accuracy. These results provide a platform for developing machine learning biosensing systems for evaluating subtle morphological changes in decellularized scaffolds, which are crucial for assessing their functionality.

## Introduction

Decellularization is, by definition, a process of removal of cell components from tissues in which vasculature and extracellular matrix (ECM) remain relatively intact. Decellularized tissues retain many of their biochemical, structural, and biomechanical properties, enabling the formation of various biologic scaffolds that act as the basis for cell growth and differentiation (Abassi et al., 2013; Corridon et al., 2017; Corridon, 2021; Corridon, 2022; Corridon et al., 2022; Wang et al., 2022a; Wang et al., 2022b). Since such tissues do not possess antigens at the cell surface, the risk of eliciting an adverse immune response is significantly reduced when transplanted to a different host. Decellularization is, therefore, today an important strategy in contemporary transplantology and regenerative medicine research (Wilson et al., 2016; Polisetti et al., 2021; Shakeel and Corridon, 2023).

Some authors state that decellularized corneas are today one of the most promising materials for the successful replication and engineering of corneal tissues, considering the complexities associated with the structural organization of its layers and extracellular matrix (Fernández-Pérez and Ahearne, 2020). Corneal decellularization is a potentially valuable approach for creating non-immunogenic scaffolds that can be later used to reconstruct primary corneal layers and subsequent transplantation (Wilson et al., 2013; Isidan et al., 2021). Unfortunately, despite numerous physical, chemical, and biological methods developed for corneal decellularization over the past decade, many unresolved issues exist in this research area (Ahearne, 2020; Fernández-Pérez and Ahearne, 2020; Holland et al., 2021). For instance, despite great success in achieving acellular scaffolds, more consensus is needed regarding the quality of the decellularized extracellular matrix 16. Some of these issues reflect the challenges associated with pathohistological and microscopy analysis of the decellularized stroma (Wilson et al., 2016; Formisano et al., 2021).

The corneal stroma is a transparent layer consisting of keratocytes and ECM components. The ECM comprises parallel bundles (fibrils) of collagen fibers that form around 200 lamellae, arranged anisotropically in a spherical coordinate system (Aldrovani et al., 2007; Meek, 2009; Sridhar, 2018). Collagen types I (predominant form), II, V, VI, XII, XIII, XIV, and XXIV, and various glycosaminoglycans can all be located in the ECM (Meek, 2009; Corridon et al., 2006; Feneck et al., 2019). Keratocytes, mesenchymal-derived fibroblast cells with complex interactions with corneal epithelium, are the most abundant cell type in the corneal stroma and have various functions regarding the synthesis and organization of the ECM (Corridon et al., 2006; West-Mays and Dwivedi, 2006). They are usually characterized by a typical dendritic morphology with cell-processes up to 50 mm long, and in some histological stains, the cells appear to be flattened and quiescent (Scott et al., 2011). Because of the relative scarcity of these cells (sometimes occupying less than 10% of the stromal volume) and their occasional blurred appearance in hematoxylin- and eosin-stained sections, it is challenging to differentiate decellularized from intact stromal tissue during the conventional microscopy analysis, compared to fluorescent approaches (Hahnel et al., 2000; Cai et al., 2022; Shaya et al., 2022). This issue is especially the case in experiments where the decellularization chemical agent has been used in low concentration and decellularization has only been partially completed.

In recent years, many new and innovative computational methods have been proposed as effective in detecting subtle alterations in tissue architecture that are otherwise difficult to notice during the standard pathohistological evaluation. Some of these approaches include textural analysis methods, such as those based on gray level co-occurrence matrix (GLCM) computations (Zaletel et al., 2021). The results of this and other methods may be used as input data for the training of numerous machine learning (ML) models, which further increases the level of automation and the sensitivity of the methods to detect changes in tissue structure. In this work, we present results indicating that the GLCM method, along with the discrete wavelet transform, can be used as an effective tool to microscopically differentiate intact corneal stromal tissue from the tissue treated with relatively low doses of the decellularization chemical agent. Furthermore, we demonstrate that it is possible to develop an ML model based on random forest and support vector machine algorithms with relatively high accuracy in classifying regions of interest in micrographs of intact and treated corneal stromal tissues.

Current histological assessments are performed in conjunction with DNA analyses to gauge the degree of decellularization. In the long-term, such algorithms may enhance decellularization characterizations by correlating the threshold concentrations of residual cellular material within the ECM that would unlikely elicit a negative remodeling response concerning the ECM source, tissue type into which the ECM is implanted, and host immune function (Crapo et al., 2011; Corridon, 2023a; Corridon, 2023b; Corridon, 2023c). Ultimately, this approach can eliminate the need for multiple-stage and time-consuming protocols and extend our ability further to examine the quality of residual ECM microstructure.

## Materials and methods

### Animals and whole eyes samples

Eyeballs from the prominent Arabian sheep breeds (Najdi, Awassi (Nuaimi), and Orb) in our region of the United Arab Emirates (UAE) were collected from a local slaughterhouse in Abu Dhabi to create native and decellularized corneal keratoprosthesis models (Khan et al., 2023). The experiments were performed with support and approvals from the Automated Slaughterhouse of the Municipality of Abu Dhabi City and the Animal Research Oversight Committee at Khalifa University of Science and Technology (Abu Dhabi, UAE) in accordance with ARRIVE criteria.

### Whole eye decellularization and corneal extraction

Cadaveric sheep eyes were excised from the ocular globe, washed several times in saline, placed in a sealed container with saline, and kept on ice in a cooler for transportation from the slaughterhouse to the laboratory. A total of 25 eyes were collected and randomly grouped into five groups, presented in Table 1. The native grafts were obtained by extracting corneas with the limbus intact and placing them in cold storage until experimentation. On the other hand, the acellular corneal scaffolds were created using whole eye immersion/agitation-based decellularization using the conditions outlined in Table 1.

**TABLE 1:** A description of the native and decellularized sample groups and decellularization conditions.

Group	Number in group	Detergent conc. (%)	Days	Decellularization condition
1	5	0	0	Native
2	5	1	2	1%2D
3	5	1	4	1%4D
4	5	4	2	4%2D
5	5	4	4	4%4D

Each cornea was placed in separate small containers and immersed in 50 mL of 1% and 4% biosurfactant solution (Ecover, Malle, Belgium) that consisted of 15%–30% non-ionic surfactants, 5%–15% anionic surfactants, ethanol, sodium citrate, glycerin, trisodium ethylenediamine disuccinate, polypropylene terephthalate, and citric acid. Each container was set up on an Ohaus™ Analog Heavy Duty Shaker (Thermo Fischer Scientific, Waltham, MA, United States) and agitated for 2 or 4 days at 300 rpm, depending on the group. After the decellularization period, the corneas were removed from the detergent solution. The corneas were then immersed in a similar volume of deionized water and agitated again at 300 rpm for 3 days to remove traces of the detergent. During the first 2 days, the water was changed at 3X daily, and then on the end of third day, the water solution was changed for a final time. After which, the corneas were sectioned for DNA and histological assessments.

### DNA quantification

DNA quantification and histological analyses experiments were performed on the extracted native or decellularized corneas to investigate the effective removal of all the cellular components using GLCM. DNA extraction was performed using the QIAamp DNA Mini kit (Qiagen, #51306, Germantown, MD, United States) per the manufacturer’s instruction (Mousa et al., 2021). Approximately 20 mg of each sample was cut into small pieces and subjected to overnight lysis. DNA was bound onto a silica membrane spin column, and further purification washes were done using wash buffers provided in the kit. Finally, DNA was eluted by using an elution buffer. Extracted DNA quantity and purity were assessed by using a Nanodrop Spectrophotometer (ThermoFisher Scientific). The quality of DNA was checked on 1% agarose gel.

## Optical transmission tests

Transmission spectra of the corneal scaffolds were obtained using a UV-Vis spectrophotometer connected to an optical microscope, Zeiss Axioscope (Zeiss Group, Oberkochen, Germany), through a fiber optic probe. The utilized spectrophotometer was USB 4000+ (Ocean Optics, Dunedin, FL, United States), which has an operation range of 200–1,100 nm, and the spectra were recorded using OceanView software. A glass slide was used as a reference for this experiment, and triplicate measurements of each sample were recorded and averaged. The spectra of the scaffolds, stored in saline, were initially measured. Then, upon placing the scaffolds in pure glycerol for 20 min, their surfaces were wiped with delicate task wipes, and their corresponding optical characteristics were recorded. The transmission spectra data were then averaged for each decellularized and native corneal sample placed in both saline and glycerol.

### Histological assessments

Corneas were fixed with 10% formalin for 1 h at 4°C. The samples were rinsed in distilled H_2_O and stored in 70% ethanol. Specimens were dehydrated through a graded series of ethanol (70%, 80%, 95%, and 100%) for 40 min each, cleared in xylene for another 40 min, infiltrated four times with paraffin for 60 min each, and finally embedded in fresh paraffin. Then Reichert-Jung 820 microtome (Depew, NY, United States) was used to cut approximately 3.5 μm thick sections that were flattened on a warm water bath and mounted on glass slides and stained with H&E. A CX43 Olympus microscope (Olympus Corporation, Tokyo, Japan) was used to acquire brightfield micrographs of the stromal regions to examine decellularization efficacy and ECM integrity. For this process, 30 images per group were collected, and 6 random areas were selected in each image to identify morphological changes in the corneal stroma that result from decellularization by outlining various regions of interest (ROIs) to separately gauge stromal fiber misalignment and damage, resulting in a score of either 0 or 1; whereby 0 represents intact (aligned or non-damaged) lamellae, whereas 1 represents impaired (misaligned or damaged) lamellae. Six ROIs were randomly selected in thirty 10X micrographs from each defined group. Three scorers blinded to the hypotheses and experimental conditions recorded the number of intact and impaired lamellae regions.

### Gray level co-occurrence matrix analysis

**FIGURE 1:**
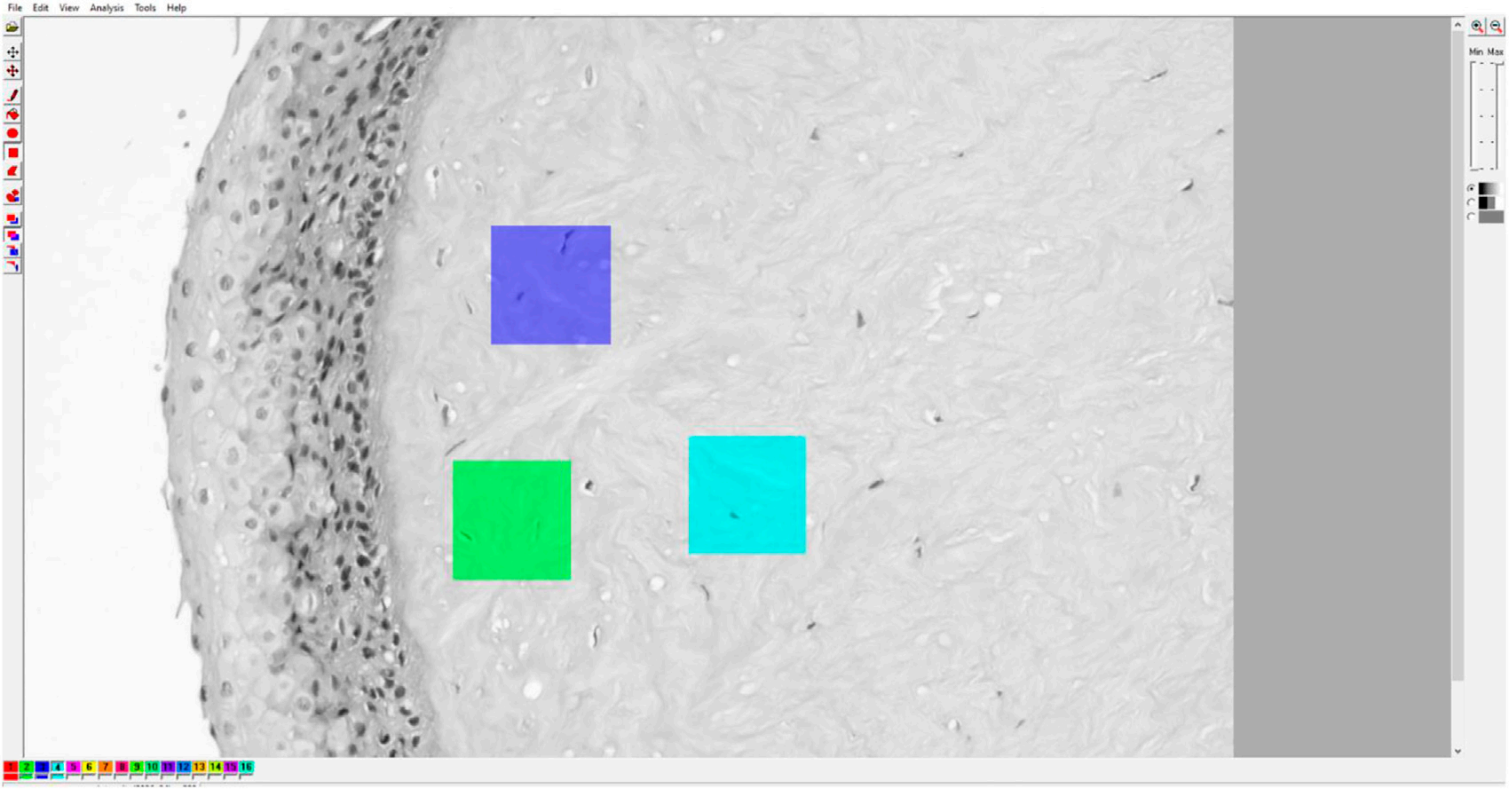
Example of Mazda user interface with a digital micrograph of native cornea. The micrographs were converted to grayscale BMP format for ROI creation and subsequent GLCM and DWT analyses.

Textural analysis was done in “Mazda” software developed and maintained by Dr. Michal Strzelecki and Dr. Piotr Szczypinski of the Institute of Electronics, Technical University of Lodz Poland (Szczypinski et al., 2007; Piotr et al., 2009; Michal et al., 2013). This software was created using C++ and Delphi^©^ programming languages within actions COST B21 European project “Physiological modelling of MR Image formation,” and COST B11 AQ6 European project “Quantitative Analysis of Magnetic Resonance Image Texture” (1998–2002). In the main part of our research, for each corneal specimen, 3 representative digital micrographs were made using 100x magnification, 3,088 × 2076 resolution units in size, with a horizontal and vertical resolution both being 96 dpi, and with a bit depth of 24 that was analyzed in a grayscale setup (Figure 1). For each digital micrograph, GLCM analysis was performed on 3 representative squared regions of interest sized 300 × 300 resolution units. For each ROI, 5 different GLCM features were quantified: Inverse difference moment (IDM), GLCM contrast (CON), GCLM correlation (COR), angular second moment (Kazim et al., 2021), and sum variance (SVAR).

It should be noted that GLCM analysis is a complex mathematical and statistical method that uses second-order statistics to calculate features from two-dimensional signals such as digital micrographs or other images. For this type of analysis, the images are first converted to grayscale format, usually 8-bit. After that, each resolution unit of the image (pixel) is assigned a numerical value based on its gray level intensity. After that, a matrix is created based on the data describing how often a specific spatial relationship occurs between a pixel with the value *i* and a pixel with the value *j*. Usually, the analyzed pixels are the immediate neighbors, or in other words, the pixel *j* is horizontally adjacent and on the right side of the pixel *i*. Second-order statistical features such as IDM, ASM and others are calculated from the frequency of occurrence of pixel pairs (i,j) that are in a predetermined spatial relationship. The features are basically an indirect way to quantify textural homogeneity (and heterogeneity), which may, in some cases, correspond to homogeneity in a morphological sense.

Considering that p(i,j) represents the (i,j)th entry of the gray-level co-occurrence matrix following the normalization, the values of ASM were calculated as:

$$ASM=\underset{i}{\sum }\underset{j}{\sum }{\left\{p\left(i,j\right)\right\}}^{2}$$

An angular second moment is often considered an indicator of textural uniformity since it is related to the uniformity of distribution of gray levels within the two-dimensional signal. Inverse difference moment is a similar GLCM feature. It represents local homogeneity of the micrograph, and in our work, was calculated as:

$$IDM=\underset{i}{\sum }\underset{j}{\sum }\frac{1}{1+{\left(i-j\right)}^{2}}\;p\left(i,j\right)$$

Textural correlation feature, which in essence is a quantification of linear dependency of gray levels, was determined as:

$$COR=\frac{\sum_{i}\sum_{j}\left(ij\right)p\left(i,j\right)-\mu_{x}\mu_{y}}{\sigma_{x}\sigma_{y}}$$

Here, the μ is the average value (mean) of GLCM rows x and y, while σ is the standard deviation. Textural contrast as a quantification of local intensity variation, and textural variance as a determinant of dispersion of gray levels around the average value, were calculated as follows:

$$CON=\underset{i}{\sum }\underset{j}{\sum }{\left(i-j\right)}^{k}P_{d}{\left[i,j\right]}^{n}$$

$$SVAR=\sum {\left[i-\sum {ip}_{x+y}\left(i\right)\right]}^{2}$$

The GLCM features were stored in the local database for ANOVA/Kruskal–Wallis statistical analysis in SPSS (v.25.0 IBM Corporation, Chicago, IL, United States).

### Discrete wavelet transform analysis

Discrete wavelet transform (DWT) analysis is sometimes used as a supplemental method to GCLM to understand better the changes occurring in textural patterns. In our study, the Mazda platform, as mentioned above, can compute wavelet coefficients (d) energies taking into consideration different filters such as high (H) and low-pass (L) during a filtering cascade of rows and columns. Such computations can be performed after the data vectors are linearly transformed into numerical ones of the same length. In our study, we quantified 3 wavelet coefficients energies, EnLH, EnHL, and EnHH, depending on the combination of filters used:

$$En=\frac{\sum_{x,y\in ROI}{\left(d_{x,y}^{subband}\right)}^{2}}{n}$$

In this equation, values of x and y are subband locations, and n is the number of resolution units in the ROI. Additional info on the DWT features can be found in our recent publications (Pantic et al., 2022; Pantic et al., 2023a; Pantic et al., 2023b), as well as in the works of other authors (Kociołek et al., 2001).

This mathematical method essentially breaks down a part of the micrograph (ROI) into various frequency components and calculates the energies of those components. By analyzing changes in these energies, one can indirectly assess textural characteristics and separate between “rough” and “smooth” textures. From our previous (unpublished) experience, when calculating DWT features in biological structures such as tissues, heterogenous cytoarchitecture usually has higher EnLH, EnHL, and EnHH. When combining DWT with the GLCM method, structures with high GLCM local homogeneity indicators, such as IDM, typically tend to have low values of wavelet coefficient energies. These features may imply that the DWT features may be good indicators of textural heterogeneity, although this remains to be confirmed by a future study.

### Machine learning models

The objective of the second part of our research was to develop an ML model capable of separating regions of interest of intact corneal stroma from the ROIs of the stromal tissue treated with the lowest dose of decellularization chemical agent (concentration of 1%, period of 2 days). We opted for 2 supervised ML approaches: random forests (RF) and support vector machine (SVM). We used the GLCM and DWT features for the input data, while the output (target) was the allocation of the ROI to the experimental or the control group of specimens. For training and testing purposes, we created and analyzed a total of 1,000 ROIs (500 of the decellularized corneal stroma and 500 of the intact stroma).

Approximately 80% of the data was used for training the model, and 20% was used for testing. The ML models were developed in Python programming language, its “Scikit-learn” programming library, which is currently open source and commercially usable). The Random forests approach an ensemble learning method that can create many different decision trees and combine their predictions to make a final prediction (Geng et al., 2022). The trees are built based on a random data subset to give a prediction on the target variable (in our case, the target was the class of the ROI describing its allocation to the experimental or control group) based on input data which are, in our case GLCM and DWT features. After all the trees are constructed, the machine model takes a majority vote to make the best prediction (the predictions from most trees are considered correct). Random forests can be used for various classification tasks when the target variable is categorical (similar to in our study) or regression tasks when the target variable is continuous (i.e., consists of data on the ratio scale). In this work, we applied the RF Scikit-learn code previously developed on micrographs of *Saccharomyces cerevisiae* cells as a part of the SensoFracTW project supported by the Science Fund of the Republic of Serbia (Pantic et al., 2023b).

The support vector machine (SVM), on the other hand, is a supervised learning algorithm based on the margin maximization principle and structural risk minimization (Adankon and Cheriet, 2015). In essence, this ML model finds the best possible boundary (or so-called “hyperplane”) that can separate two classes of data points (in our case, the points belonging to experimental and control groups). The goal of this classifier would be to maximize the margin (distance) between the boundary and the nearest data points. For the SVM to achieve this, the data points must be mapped to a higher-dimensional space, usually using a specialized “kernel” mathematical function. In our study, the Python and “Scikit-learn” codes for both models were implemented in the Jupyter Notebook service hosted in “Colaboratory,” a product developed by Google Research. Using this approach, we also quantified the performance of the models by calculating their classification accuracy and the area under the receiver operating characteristic curve (discriminatory power).

In addition to the computational analysis described above, we performed a subjective classification of the corneal stroma regions of interest. Individuals with comprehensive experience in histological evaluations were presented with 500 ROIs of the decellularized corneal stroma (obtained using a 1% concentration of the decellularization chemical agent for 2 days) and 500 ROIs of the intact stroma. These ROIs were randomly selected and were similar to those used for the training and testing of ML models, following the selection protocol described in a recently published work (Pantic et al., 2023a). Briefly, the ROIs were sized to 300 × 300 resolution units using the ImageJ software platform (National Institutes of Health, Bethesda, MD) and then cleared of the surrounding area (ImageJ > Edit > Clear Outside). The observer assigned a value of “0” or “1” based on their subjective assessment of whether the ROI belonged to the control or experimental group. After allocating the values, the actual classes of the ROIs were revealed, and a ROC analysis of the “subjective method” was performed.

## Results

### DNA assessment of corneal decellularization

**FIGURE 2:**
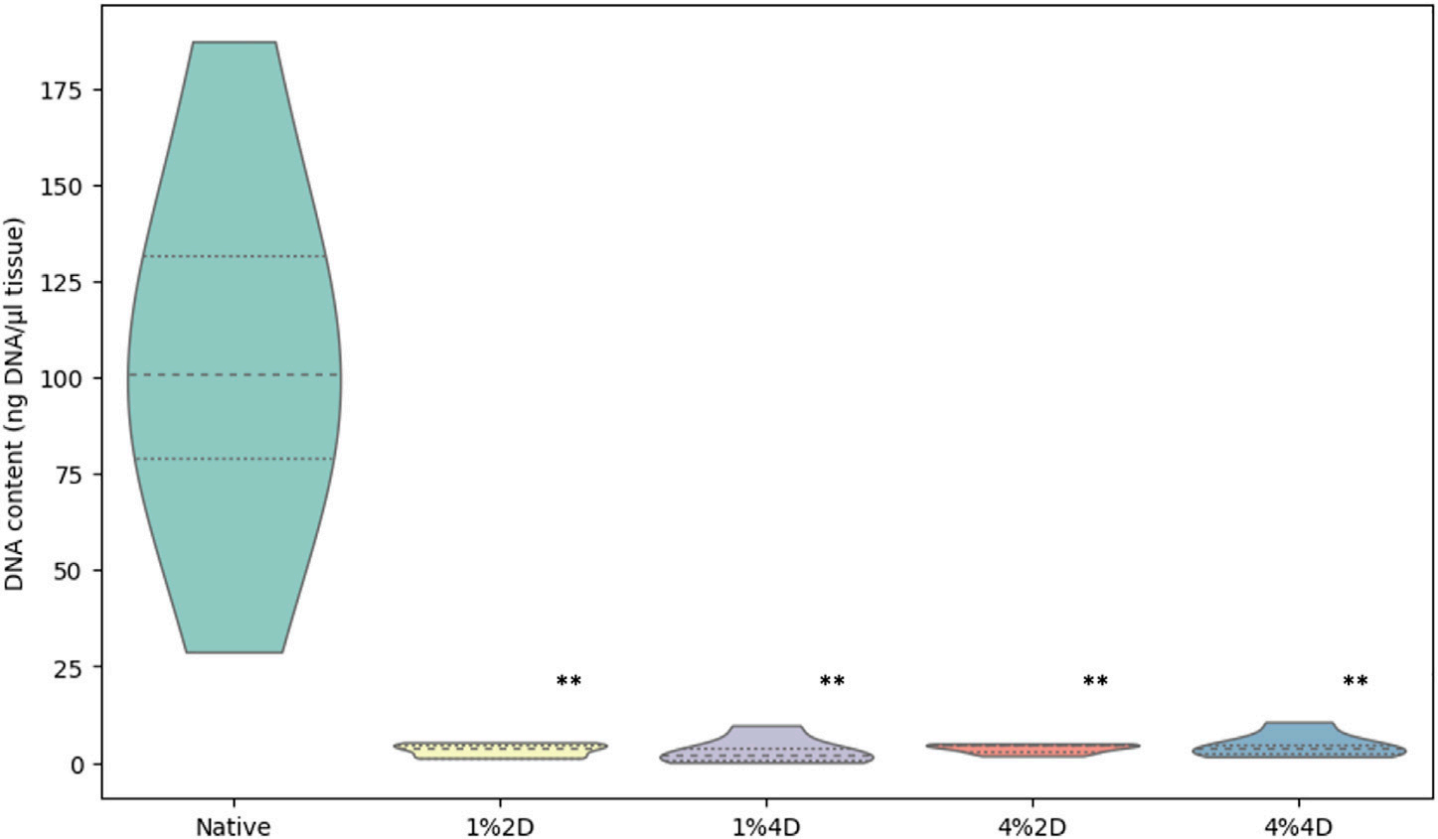
Light transmittance through native and decellularized corneas. Comparison of DNA contents in native and acellular corneas. Residual DNA contents estimated in scaffolds produced from each decellularization condition significantly differed from the DNA contents in the native corneas. The *p* values are based on the Kruskal–Wallis test that identified the difference among the groups (*p* < 0.002), and pairwise group comparisons generated using the Dunn’s *post hoc* test highlight the significant difference between the native DNA concentration and DNA concentration retained after each decellularization approach (**), before and after the Bonferroni correction.

The residual DNA in each decellularized cornea was quantified and compared to the DNA content in native corneas to examine the effectiveness of each decellularization approach. All four conditions removed an average of more than 95% of native DNA, a benchmark indicating effective cell removal outlined by previous reports (Caralt et al., 2015). The residual DNA contents are shown in Figure 2 and represent significant reductions using each condition, which were revealed with the Kruskal–Wallis (*p* < 0.002). From the data, we observed a 97% reduction in DNA contents in scaffolds from group 1%2D (*p* = 0.003 and p^adj^ = 0.028) and group 1%4D (*p* < 0.001 and p^adj^ = 0.004), a 96.4%, reduction (*p* < 0.007 and p^adj^ = 0.072) in scaffolds form group 4%2D, and 95.6% reduction (*p* < 0.007 and p^adj^ = 0.067) in scaffolds from group 4%4D compared to native tissues after applying the Dunn’s pairwise test. The term p^adj^, denotes the *p* values obtained for the pairwise test after applying the Bonferroni correction, which only identified pairwise comparisons, native-1%2D, and native-1%4D, as significant. These results are summarized in Table 2.

**TABLE 2:** A summary of statistical analyses used to estimate significant reductions in DNA contents observed with each decellularization condition.

Group comparisons	Native and decellularization conditions	% reduction in DNA content	*p*-value	Adjusted *p*-value
Group 1 vs Group 2	Native vs 1%2D	97.0	0.003	0.028
Group 1 vs Group 3	Native vs 1%4D	97.0	<0.001	0.004
Group 1 vs Group 4	Native vs 4%2D	96.4	<0.007	0.072
Group 1 vs Group 5	Native vs 4%4D	95.6	<0.007	0.067

### Transmittance through native and decellularized corneas

The degrees of optical transmittance from our reference (a transparent glass slide) and native and decellularized corneas with and without glycerol treatment are presented in Figure 2. The Kruskal–Wallis test revealed a significant difference (*p* < 0.002) among the measured groups. Decellularized corneas treated with glycerol are shown in Groups 6–9; the reference is Group 10. These measurements confirmed that the reference (control) was almost 100% transparent, while the native was nearly 55% transparent, and the scaffolds before glycerol treatment facilitated roughly 0%–3% light transmission. Conversely, glycerol treatment reversed corneal opacity and substantially enhanced light propagation through these sections, supporting approximately 40%–50% degrees of light transmission. Likewise, the Kruskal–Wallis test revealed that the degrees of optical transmittance among the groups (*p* < 0.002), as well as significant pairwise differences, except for the data obtained compared from scaffolds treated by 1%2D and 4%4D (presented in Supplementary Table S1).

### Macroscopic and histological assessments

**FIGURE 3:**
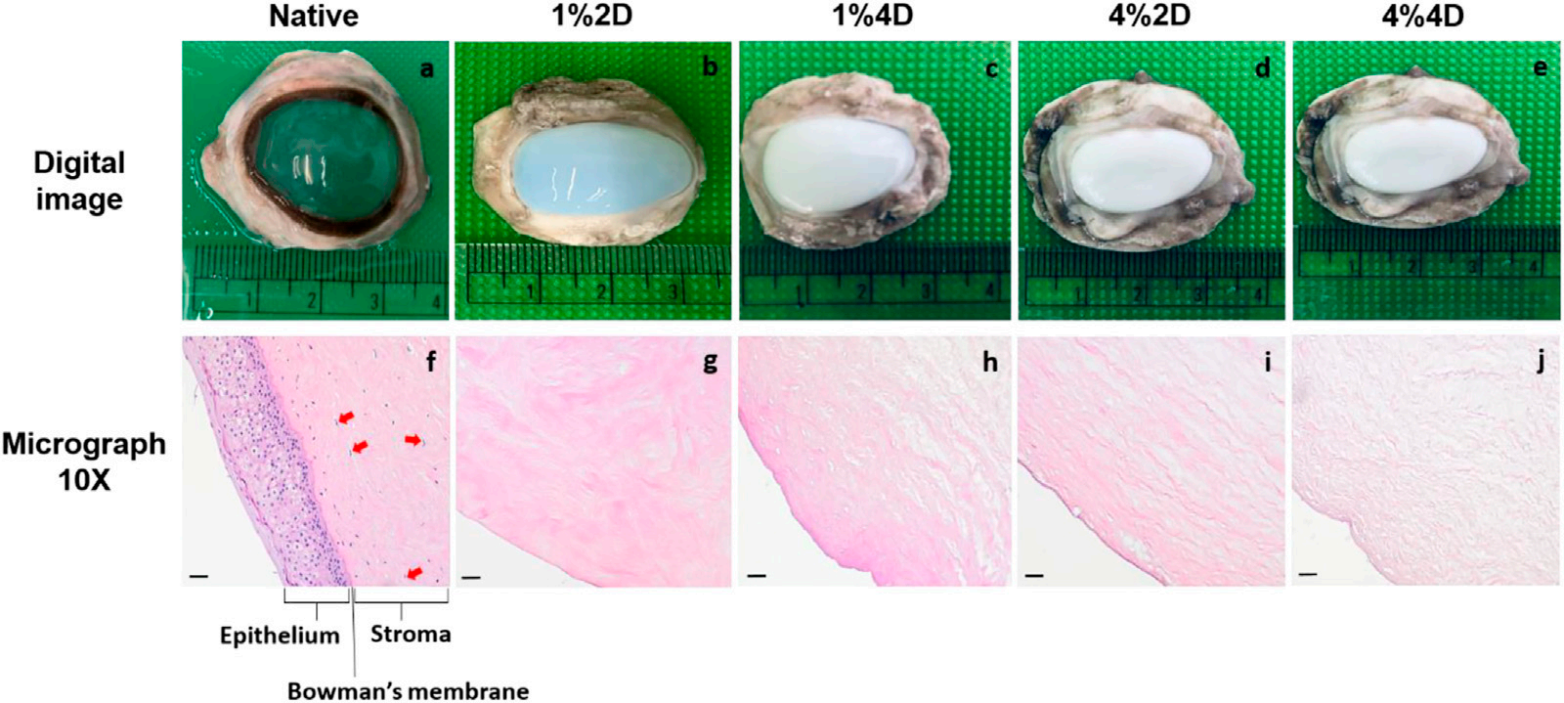
Digital images and brightfield 10X micrographs of native and decellularized ovine corneas. Digital images of an extracted **(A)** native cornea (group 1), **(B)** cornea decellularized using 1% biosurfactant for 2 days (group 1), **(C)** cornea decellularized using 1% biosurfactant for 4 days (group 1), **(D)** cornea decellularized using 1% biosurfactant for 2 days (group 1). Brightfield 10X micrographs obtained from an extracted **(A)** native cornea (group 1), **(B)** cornea decellularized using 1% biosurfactant for 2 days (group 1), **(C)** cornea decellularized using 1% biosurfactant for 4 days (group 1), **(D)** cornea decellularized using 1% biosurfactant for 2 days (group 1). The epithelium, Bowman’s membrane, and stroma (specifically with the presence of keratocytes identified by the arrows) are clearly defined in native tissues compared to the decellularized scaffolds. Scale bars = 20 μm. Digital images and brightfield 10X micrographs using 1% biosurfactant for 2 days (group 2), **(C)** cornea and **(E)** cornea decellularized using 4% biosurfactant using 1% biosurfactant for 2 days (group 2), **(H)** cornea and **(J)** cornea decellularized using 4% biosurfactant the arrows) are clearly defined in native tissues compared.

Regardless of the initial decellularization condition, all the biosurfactant-treated ovine corneas became opaque, in varying degrees (Figure 3B through Figure 3E) compared to native transparent corneas (Figure 3A), which is a visible hallmark of corneal decellularization (Wilson et al., 2016; Polisetti et al., 2021). Furthermore, histological assessments were conducted on acellular scaffolds and native corneal tissues to examine the quality and extent of decellularization, as well as the effect each condition had on the stromal ECM. These assessments identified each decellularizing condition’s ability to effectively balance cellular removal and retention of the innate ECM architecture, albeit to different extents. In each case, micrographs obtained from scaffolds illustrated the absence of cellular components throughout corneal compartments, using each decellularized condition (Figure 3G through Figure 3J), compared to the native corneal layers (Figure 3F). These results correlate well with our DNA-based biochemical assays.

**FIGURE 4:**
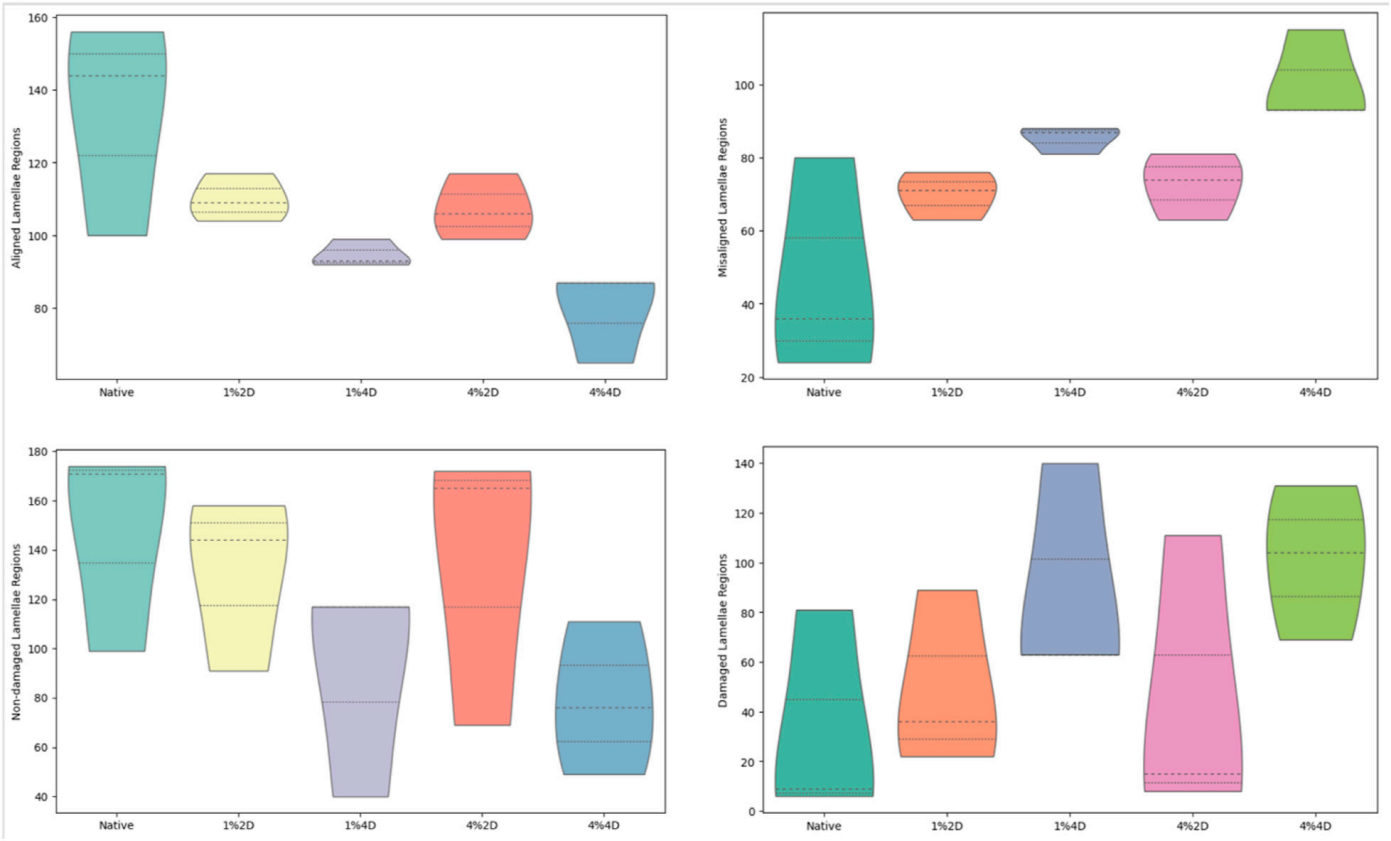
Histological assessments identifying morphological fiber misalignment and damage that resulted from decellularization.

However, the quality of the resulting ECM varied for different decellularization conditions as we estimated the morphological changes in the corneal stromal lamellae based on decellularization concentration and duration. These micrographs showed that decellularization concentration had a greater effect than duration. Specifically, collagen layers appeared more ordered and stacked after treatments with the lower (1%) biosurfactant concentration. In contrast, the higher biosurfactant concentration appeared to produce more disruptions to the intrinsic lamellae structure (Figure 4). These blinded semi-quantitative analyses also revealed notable variations in the degrees of intactness pertaining to lamellae alignment and damage.

Each scorer examined a total of 900 images, i.e., 180 images for each of the 5 treatment groups. From these assessments, the Kruskal–Wallis test revealed statistically significant differences only among the numbers of aligned (*p* = 0.025) and misaligned (*p* = 0.025) stromal lamellae. For the statistical evaluation of numbers of aligned stromal lamellae, the Dunn’s adhoc test outlined pairwise differences between 4%4D-4%2D (*p* = 0.035 and padj = 0.353), 4%4D-1%2D (*p* = 0.005 and padj = 0.153), 4%4D-native (*p* = 0.005 and padj = 0.045), and 1%4D-native (*p* = 0.049 and padj = 0.491). Similarly, for the statistical assessment of numbers of aligned stromal lamellae, the Dunn’s adhoc test outlined pairwise differences between 1%4D-native (*p* = 0.049 and padj = 0.491), 4%4D-native (*p* = 0.005 and padj = 0.045), 1%2D-4%4D (*p* = 0.015 and padj = 0.153), 4%2D-4%4D (*p* = 0.035 and padj = 0.353).

### Gray level co-occurrence matrix and wavelet analysis

**FIGURE 5:**
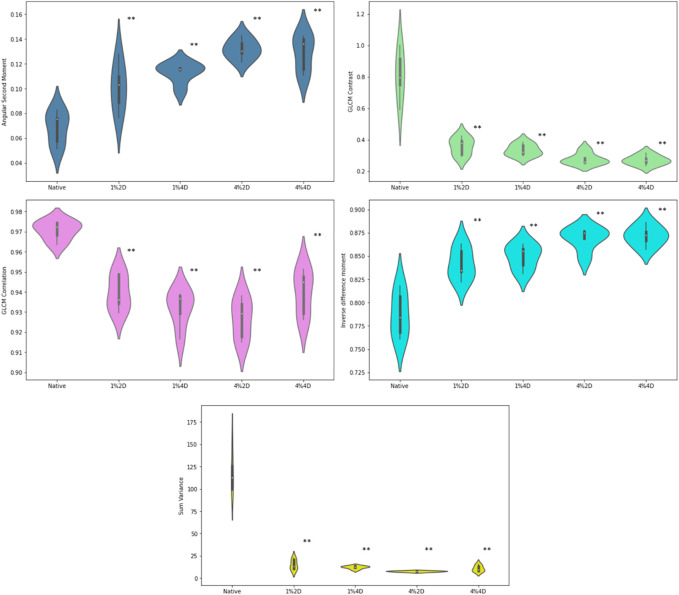
GLCM indicators for native corneas and scaffolds created using various decellularization conditions. **p < 0.01 compared to controls.

GLCM indicators for native corneas and scaffolds created using various decellularization conditions are presented in Figure 5. From these plots, the mean value and standard deviation of the angular second moment of corneal stroma were 0.068637 ± .01337 in the control group. It was significantly increased in all experimental groups (*p* < 0.001). In the first (treatment with 1% of decellularization agent, for 2 days), second (1% for 4 days), and third (4% for 2 days) experimental group, the values were 0.101 ± 0.019, 0.113 ± 0.008 and 0.132 ± 0.008, respectively, while in the fourth group (treatment with 4% of decellularization agent, for 4 days) there was a slight non-significant reduction compared with the third group (0.129 ± 0.015) but still highly significant when compared with to the controls. From this finding, we could conclude that decellularization, even with a relatively small concentration of the agent, causes a substantial increase in textural homogeneity of the corneal stroma, which can be quantified using the gray level co-occurrence matrix method.

The average values in the four experimental groups were 0.842 ± 0.017, 0.849 ± 0.013, 0.869 ± 0.012 and 0.871 ± 0.011, and all of the values were significantly (*p* < 0.001) increased in comparison to the controls (0.787 ± 0.024). We could observe a relatively stable and significant trend in this increase, indicating the dose dependence on the chemical agent. Similar results were observed in the changes of local textural homogeneity quantified with inverse difference moment.

For the values of the textural contrast and GLCM correlation feature, an opposite trend was observed compared to ASM and IDM. The mean values of the textural contrast experimental groups were 0.360 ± 0.055, 0.336 ± 0.037, 0.278 ± 0.036 and 0.271 ± 0.031, respectively, which represented a substantial reduction (*p* < 0.001) when compared to the control group (0.810 ± 0.157). The average value of correlation in the control group was 0.971 ± 0.005, and it significantly (*p* < 0.001) decreased in experimental groups to 0.940 ± 0.009, 0.931 ± 0.009, 0.927 ± 0.010 and 0.940 ± 0.011, respectively. A similar but even more drastic reduction was observed in the average values in textural sum variance, which in the controls equaled 117.75 ± 22.37. In contrast, in the experimental groups, it equaled 15.15 ± 5.70, 12.20 ± 1.72, 7.57 ± 0.69, and 10.80 ± 3.52, respectively.

**FIGURE 6:**
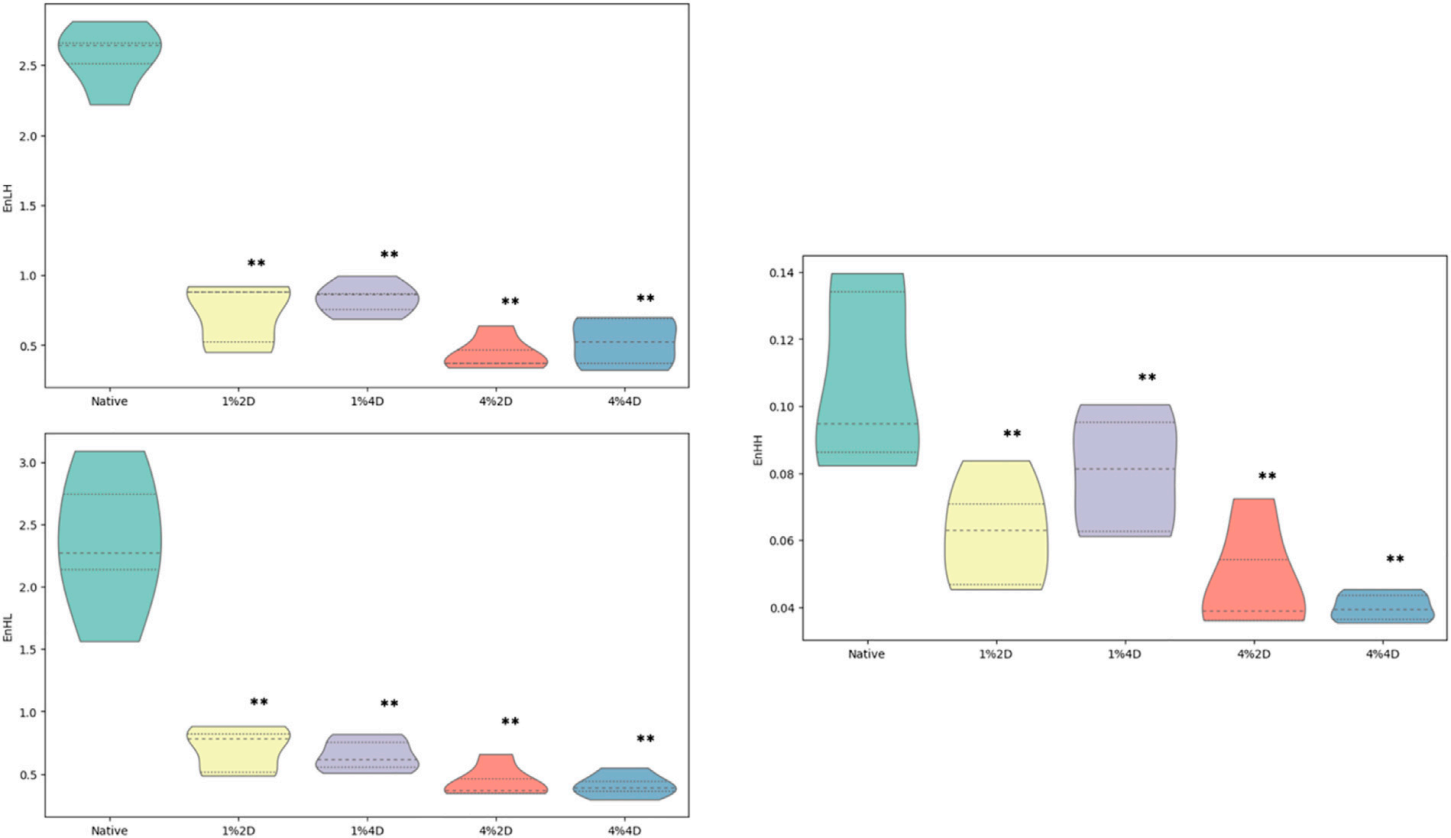
Mean values of discrete wavelet transform coefficient energies. **p < 0.01.

After decellularization, we observed a statistically significant reduction in all quantified wavelet coefficient energies (Figure 6). The mean value of the WavEnLH feature in experimental groups was 0.732 ± 0.224, 0.834 ± 0.118, 0.440 ± 0.122, and 0.524 ± 0.175 which was significantly lower than its average value in controls (2.570 ± 0.223, *p* < 0.001). A similar reduction was detected for the WavEnHL indicator, with the mean values in experimental groups equaling 0.699 ± 0.183, 0.651 ± 0.132, 0.438 ± 0.132 and 0.410 ± 0.095, while in controls, it was 2.362 ± 0.585 (*p* < 0.001). Finally, the WavEnHH feature also significantly decreased from the value of 0.107 ± 0.027 in the control group to the values of 0.062 ± 0.016, 0.080 ± 0.018, 0.048 ± 0.016, and 0.040 ± 0.004 in experimental groups.

**FIGURE 7:**
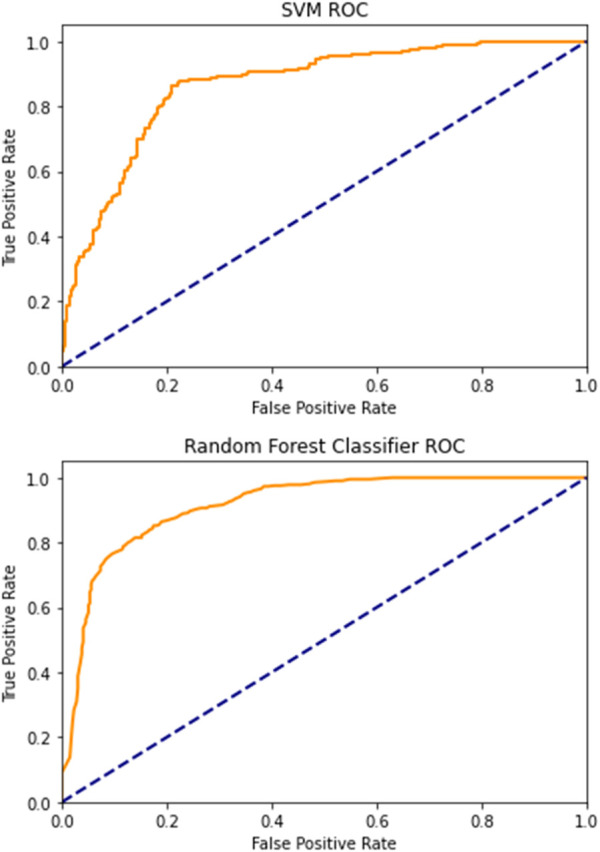
Receiver operating characteristics curves for SVM and RF ML models.

Regarding the ML models, we obtained the best performance when for input data, we used Inverse difference moment, GLCM contrast, GCLM correlation, angular second moment and variance, EnLH, EnHL, and EnHH. After performing 5-fold cross-validation on the training data using “cross_val_predict” function in Scikit-Learn, the estimated accuracy of the random forests model was 82.67%, while the area under the receiver operating characteristics curve was approximately 0.92. The classification accuracy of the support vector machine was 80.5% which was relatively similar to random forests. However, the support vector approach had significantly lower discriminatory power in separating treated stromal ROIs from intact stromal ROIs with the area under the receiver operating characteristics curve of 0.84 (Figure 7). It was concluded that random forests were a more powerful approach for evaluating morphological changes in corneal stroma associated with decellularization, although support vector machines also presented a satisfactory performance.

It should be noted that the wavelet indicators, when analyzed separately, also showed relatively high classification accuracy and discriminatory power. The most important feature was EnHL, with an area under the ROC curve of 0.79, followed by the EnHH feature, with a calculated area of 0.78. The wavelet coefficient energy EnLH was associated with slightly lower discriminatory power, with an area under the curve of 0.76. Although the RF and SVM models combining GLCM and DWT input data were significantly more powerful, this finding implies the potential value of DWT as a stand-alone computational method applicable in corneal pathology research.

After performing the classification of corneal ROIs based on subjective observer assessment, it was found that the classification accuracy of this approach was 60.8%, which was considerably lower when compared to the accuracy achieved by the developed ML models. The area under the receiver operating characteristic curve was only 0.61, indicating poor discriminatory power in separating ROIs from the decellularized and intact corneal stroma. These findings suggest that individual GLCM and DWT indicators, as well as SVM and RF models trained based on these data, hold some scientific value regarding their ability to identify decellularized corneal tissue.

## Discussion

Surfactants used for corneal decellularization have mainly been ionic (such as sodium dodecyl sulfate, SDS) and non-ionic (Triton X-100) (Isidan et al., 2019). Similarly, zwitterionic surfactants, like the most widely used agent of this type, 3-((3-cholamidopropyl) dimethylammonio)-1-propanesulfonate (CHAPS) have also been effective in isolating the corneal ECM from cellular components (Marin-Tapia et al., 2021). Other reports have shown that neutral hydrophilic groups of zwitterionic detergents can protect native proteins during decellularization (Wang et al., 2022a). Using these arguments as a basis for our study, we investigated how the quality of the decellularized corneal ECM is affected by zwitterionic surfactant concentration and processing time.

This work shows that effective decellularization was achieved with a zwitterionic biosurfactant, as evidenced by a greater than 95% cellular removal efficiency and corneal opacity, which can be restored using an optical clearing agent like glycerol (Polisetti et al., 2021). Furthermore, histological analysis showed subtle alterations in the lamellae structure that varied according to decellularization concentration and processing time, consistent with the previous reports (Luo et al., 2019; Polisetti et al., 2021). Such alterations compromise the biomechanical and bio-inductive properties of the scaffolds (Williams, 2019), as well as limit their optical transmission. Typically, analyses used to examine the quality of residual ECM microstructure require user-driven, multiple-stage, and time-consuming protocols. As a result, computational-based approaches may help resolve these issues.

From our investigations, we realized that exposing corneal stromal tissues to a decellularizing chemical agent leads to significant changes in the features of gray level co-occurrence matrix and discrete wavelet transform, as with conventional histological scoring. These changes indicate a substantial increase in textural uniformity and local homogeneity and a reduction of stroma’s co-occurrence and wavelet heterogeneity, probably due to the loss of keratocytes and their nuclei. In this study, we also demonstrate that it is possible to develop and test ML models based on support vector machine and random forests using GLCM and DWT as input data, which will substantially decrease evaluation time and complexity as evidenced using multiple scorers that each required at least 2 days to perform these assessments. These models presented good performance indicators, and their classificational accuracy and discriminatory power in differentiating regions of interest of treated and intact stroma were relatively high. To the best of our knowledge, this is the first research to combine knowledge and techniques of decellularization with computational algorithms such as GLCM and DWT and to use the raw data to propose a contemporary ML model based on supervised ML (Schmitt et al., 2017).

Gray level co-occurrence analysis of the tissue texture is a frequently used method to quantify subtle alterations of tissue architecture that occur after inducing damage by a chemical, physical or biological agent. One of the first works to use GLCM for this purpose was the research by Shamir et al. (2009) (Shamir et al., 2009), where this method was used to evaluate the level of structural deterioration of the muscle tissue on experimental animal model and to relate it to gene expression. Later, GLCM analysis of tissue micrograph ROIs was successfully applied to separate different areas of white mass in the central nervous system (Pantic et al., 2014). Textural analysis with GLCM was also used to prove that structurally similar layers of the hippocampus can be distinguished during computational evaluation (Pantic et al., 2015). Over time, the method also proved helpful in assessing micrographs of cancer tissue stained with the conventional hematoxylin-eosin (H&E) technique (Kolarević et al., 2018). A particularly important application of GLCM is in evaluating changes in chromatin distribution within nuclei of individual cells (Lee et al., 2021). The GLCM-quantified reorganization of chromatin patterns during pathological processes such as carcinogenesis may have some diagnostic and prognostic value in various medical disciplines.

At present, there are relatively few works in which GLCM data is used to develop ML models. Most of these models are used in radiology to classify or predict different phenomena related to the presence of tumor tissue (Reddy et al., 2019; Saju et al., 2022). One of the rare approaches where GLCM is used in light microscopy analysis is our recent work in yeast cells exposed to sublethal doses of ethanol intended to induce low-level damage (Davidovic et al., 2022; Pantic et al., 2023c). Here we compared the ML algorithms based on a multilayer perceptron neural network, decision trees (random trees), and binomial logistic regression. Although all three models had similar performance in terms of classification accuracy and area under the ROC curve, it seems that the multilayer perceptron may be the best approach for the analysis of individual nuclei in these conditions.

Regarding the application of the GLCM method in the assessment of changes occurring in the corneal tissue, to the best of our knowledge, in the past, only one focused on corneal stroma (Marian et al., 2013). In that study, image texture analysis was used to evaluate changes in corneal surface roughness, and the tissue was examined using a scanning electron microscope. Surface roughness was assessed subjectively and using second-order GLCM statistics. The authors concluded that compared to the subjective manual assessments method, and is also objective, quantitative, faster, and more robust. However, the results are not directly comparable with our current findings due to significant methodological differences (e.g., microscopy technique, aspects related to the GLCM method, etc.).

The computational method is valuable in designing computer-aided diagnostic systems for corneal diseases that use topographical parameters to increase the probability of making the correct clinical decision. A recent study utilized the GLCM method to analyze three-dimensional corneal maps obtained from a non-invasive imaging device using Scheimpflug imaging technology (Cengiz, 2021). In addition, the authors proposed supervised ML approaches, which included Naive Bayes, decision trees, support vector machines, and the K-Nearest Neighbors algorithm. These models were trained on a relatively large dataset of more than 4,000 corneal maps and provided a reasonable basis for further ML development in corneal pathology.

The computational method is valuable in designing computer-aided diagnostic systems for corneal diseases that use topographical parameters to increase the probability of making the correct clinical decision. A recent study utilized the GLCM method to analyze three-dimensional corneal maps obtained from a non-invasive imaging device using Scheimpflug imaging technology (Cengiz, 2021). In addition, the authors proposed supervised ML approaches, which included Naive Bayes, decision trees, support vector machines, and the K-Nearest Neighbors algorithm. These models were trained on a relatively large dataset of more than 4,000 corneal maps and provided a reasonable basis for further ML development in corneal pathology.

Regarding applying the GLCM method in the analysis of light microscopy images, a similar approach combining textural analysis with ML was used in a recent study on laryngeal cancer micrographs (Valjarevic, 2023). GLCM and DWT methods were used to detect subtle changes in the nuclear structural organization of squamous epithelial cells in cancer biopsies. This process enabled the researchers to distinguish them from morphologically similar cells in benign chronic laryngitis. Like our current study, random forests and support vector ML algorithms were trained to differentiate between the nuclear regions of interest. In this case, the classification accuracies of the support vector machine and random forest models were 81% and 84.5%, respectively. The random forest also outperformed the SVM in other indicators, such as the area under the ROC curve.

Finally, one should mention a recent study on applying GLCM and DWT methods in assessing microscopic kidney structure after mild acute injury (Pantic et al., 2023a) and ways to examine the integrity of the decellularized renal structure (Pantic et al., 2022). In the first study, textural analysis was performed on proximal tubule cells after inducing mild damage under experimental conditions, after which nuclear ROIs were evaluated computationally. It was indicated that GLCM and DWT could identify subtle alterations in cell structure that are not visible using subjective methods. Similarly, to our current research, the personal evaluation and classification of the ROIs were performed, and it was determined that ML methods have far greater classification accuracy and discriminatory power. In addition, to support vector machine and random forest, a model based on binomial logistic regression was also developed and showed comparable performance, suggesting that this approach may also be considered in future research where GLCM and DWT features are used as ML input data. In the second study, we applied the GLCM computational algorithms to analyze decellularized porcine kidneys’ vascular and parenchymal integrity under various physiologically relevant perfusion conditions. This study revealed the ability to generate statistically significant changes in GLCM and wavelet features, including the reduction of the angular second and inverse difference moments, which indicated substantial increases in angiographic textural heterogeneity in a manner that augments conventional fluoroscopic angiography analyses of micro/microarchitectural integrities.

Additionally, from a functional perspective, optical transmission tests showed a substantial variation in the rates of visible light transmitted through decellularized corneas compared to native corneas, which can be attributed to the experimental conditions. Corneal opacity in native tissues can be generated through tissue extraction and storage processes, which are heightened with decellularization. Overall, multiple scattering layers in biological tissues limit optical penetration depth and hence low transmission. The tissue components (including cells, membranes, collagen, fibrin, and elastin fibers) have refractive indices from 1.47 to 1.51, while the surrounding saline solution has a refractive index of nearly 1.3364,65. This mismatch between the refractive indexes creates interferences from which light diffuses and scatters. Index matching can be achieved by using a solvent with a higher refractive index, such as glycerol, with a refractive index of 1.4766,67, reducing the optical inhomogeneity in the tissue samples. As shown in Figure 2D, the index-matching agent glycerol facilitated the reversal of ocular opacity (Figure 1) and enhanced the degree of light transmittance in these xenografts.

Additionally, from a functional perspective, optical transmission tests showed a substantial variation in the rates of visible light transmitted through decellularized corneas compared to native corneas, which can be attributed to the experimental conditions. Corneal opacity in native tissues can be generated through tissue extraction and storage processes, which are heightened with decellularization. Overall, multiple scattering layers in biological tissues limit optical penetration depth and hence low transmission. The tissue components (including cells, membranes, collagen, fibrin, and elastin fibers) have refractive indices from 1.47 to 1.51, while the surrounding saline solution has a refractive index of nearly 1.33 (Costantini et al., 2019; Hamdy and Abdelazeem, 2020). This mismatch between the refractive indexes creates interferences from which light diffuses and scatters. Index matching can be achieved by using a solvent with a higher refractive index, such as glycerol, with a refractive index of 1.47 (Bochert et al., 2005; Hedhly et al., 2022), reducing the optical inhomogeneity in the tissue samples. Overall, this index-matching agent facilitated the reversal of ocular opacity and enhanced the degree of light transmittance in these xenografts.

Our transmittance studies allowed us to examine the degrees of optical light transmission through the decellularized corneas to the native cornea. Specifically, the transmission spectra of the corneal scaffolds were obtained using a UV-Vis spectrophotometer. The approach allowed us to gauge the functional aspects of the scaffolds, and compared with our previous studies (Xinyu Wang and Salih, 2023). Other studies have also reported that decellularized scaffolds, generated from detergent-based procedures comparable to ours, have been recellularized using stem cells and corneal-derived cells (Fernández-Pérez and Ahearne, 2020; Polisetti et al., 2021). Such studies can be classified according to the corneal layer of interest, namely, the epithelial, stromal, and endothelial layers, and the cell source, including stem (adipose-derived and induced pluripotent forms), as well as epithelial, keratocytes/corneal Fibroblasts, and endothelial cells. These studies’ promising results highlight the opportunity for autologous and allogeneic cell transplantation. Major determinants that drive recellularization relate to the effective removal of remnant cellular components, retention of ECM compartments, and removal of the decellularization agent. Our scaffolds adhere to these criteria, and thus we believe they can support recellularization practices, which will be examined in future studies.

From a computation perspective, our results’ potential value reflects that GLCM and DWT indicators of corneal stroma changed drastically even when a relatively small dose of the decellularizing agent was applied. Given that normal corneal stroma is generally deprived of cells and mainly composed of extracellular matrix (sometimes more than 90% of the volume), GLCM and DWT methods can detect even minor alterations in textural patterns associated with cell loss. With the addition of ML as a way to automate these methods, these methods may contribute to the future development of sensing systems for evaluating decellularized tissue and estimating the efficiency of chemical substances used to remove cell components from the tissue. Of course, one should know that our results are only preliminary and must be confirmed in different conditions using different staining and microscopy protocols.

Despite the apparent advantages of gray-level co-occurrence matrix and discrete wavelet transform algorithms, they have several limitations that may hinder their applications in pathology, histology, and other areas of medicine. First, the consistency of results across different computational platforms may need to be clarified since the coding behind the software is often based on different programming languages and approaches. For example, the values of GLCM quantifications obtained on Mazda software may differ from those obtained from other texture analysis programs that also claim to calculate the same textural indicators. Second, it is known that textural features may greatly vary depending on the applied staining technique. The conventional H&E stain will probably yield different GLCM results than other stains, such as those applied during immunohistochemistry. Finally, sometimes very discrete changes in experimental conditions may significantly influence final results. For example, minor modifications in the sharpness of the visual field during light microscopy or changes in light exposure and brightness may change the resulting textural features in the micrograph. The extent to which these changes impact textual analyses is yet to be well studied.

Regarding the ML models, one has to stress the potential importance of the ML in future diagnostic pathology protocols as these approaches may greatly contribute to the automation, objectivity, and reduction of diagnostic errors. However, ML models applied in our study have significant limitations that may reduce their overall scientific value. First, the ROI sample used for training was relatively low, and a more considerable amount of training data would significantly increase the performance. Second, these models are trained to classify ROIs and are not intended to be applied to conditions where a micrograph or a tissue sample is regarded as a statistical unit of measurement. This issue dramatically limits their applicability in future work in transplantation and regenerative medicine since, in practice, experts in this field deal with tissue specimens (Corridon et al., 2013; Hall et al., 2013; Collett et al., 2017; Kolb et al., 2018; Corridon et al., 2021) rather than ROIs. Nevertheless, to the best of our knowledge, this is the first study to demonstrate that creating such ML models is possible. The obtained results may be helpful as a starting point for the future development of AI-based biosensors for evaluating decellularized corneal tissue.

In the future, deep learning algorithms could be applied to bridge the current gap in knowledge between ML in corneal pathology and actual artificial intelligence systems. Deep learning models, particularly those based on multilayer perceptrons and convolutional neural networks (CNNs), can learn features directly from raw image data and pixel values, leading to more accurate and efficient predictions. It may be possible to train these neural networks to calculate GLCM and DWT features and use them to train other networks for image or ROI classification. Additionally, CNNs could be trained to identify different complex patterns and features in corneal digital micrographs, further improving classification accuracy and discriminatory power compared to other ML methods, such as random forests and support vector machines. However, it should be noted that applying deep learning models would require a significantly larger dataset, and hyperparameter tuning and testing various architectures of neural networks would require significant computational resources not typically available in standard laboratory settings. Nevertheless, deep learning methods offer considerable potential for improving ML approaches to identify regions of interest in acellularized corneal stromal tissue.

In the future, we estimate that comprehensive quality assurance must be performed for both GLCM and DWT methods regarding their use in pathology and histology. I recommend creating a standardized computing protocol that controls all confounding factors that could influence the final results. This approach would have to be at least partially focused on testing the inter-observer reliability to see if multiple observers can obtain/calculate the textural quantifications the same way. Also, the variability of the GLCM calculations across different software platforms will have to be considered. The same applies to the variability of the features when other histological protocols are implemented.

Regarding ML applications, additional models need to be trained and tested in the future. Such procedures include supervised learning approaches such as neural networks, Naive Bayes, Linear discriminant analysis, and K-nearest neighbor algorithms. Finally, combining techniques commonly used for computer vision and image recognition with GLCM and DWT data also remains a potential option for improving the classification and prediction of biological phenomena associated with decellularization.

## Conclusion

As expected, increasing the decellularization agent concentration and duration causes more disruptions to the ECM. We could detect significant differences between decellularization conditions using our semi-quantitative and automated approaches in certain situations. Our results indicate that the decellularization in corneal stroma leads to substantial changes in indicators of gray-level co-occurrence matrix and discrete wavelet transforms. The detected changes are distinct even when using relatively small concentrations of a decellularization chemical agent, suggesting the potential scientific value of GLCM and DWT methods in pathology, ophthalmology, and regenerative medicine. Such automated detection schemes can help optimize decellularization protocols. Minimizing the zwitterionic surfactant concentration and the processing time is beneficial to improve the decellularized ECM’s quality. To the best of our knowledge, this represents the first work on using GLCM and DWT computational techniques to assess corneal stromal architecture after decellularization. We also demonstrate that it is possible to develop contemporary ML models based on random forests and support vector machine algorithms capable of identifying regions of interest in decellularized corneal stromal tissue with relatively high accuracy. The results may be a valuable foundation for the future design of innovative and efficient biosensing systems for evaluating corneal morphological changes associated with physiological and pathological processes.

## Data Availability

The raw data supporting the conclusions of this article will be made available by the authors, without undue reservation.

## In Memoriam

It is with deep sadness and heavy hearts that we recognize the passing of our colleague and friend, Georg A. Petroianu, who passed away on 28th January 2023. Georg passed away unexpectedly after a brief illness. We are so grateful for the time we shared with him and the work we achieved, and we dedicate this manuscript to Georg.
