# Supplementary material for: Computational approaches for evaluating morphological changes in the corneal stroma associated with decellularization
Source: Front Bioeng Biotechnol. 2023 May 26;11:1105377. doi: 10.3389/fbioe.2023.1105377 (PMC10250676; doi:10.3389/fbioe.2023.1105377)
Supplement: Supplementary file 1 [file Table1.DOCX]

| Group Comparisons | Native, Decellularization, and glycerol treatment | p-value | Adjusted p-value |
| --- | --- | --- | --- |
| Group 4 vs. Group 3 | 4%2D vs. 1%4D | <.001 | 0 |
| Group 4 vs. Group 2 | 4%2D vs. 1%2D | 0 | 0 |
| Group 4 vs. Group 5 | 4%2D vs. 4%4D | 0 0 | 0 |
| Group 4 vs. Group 9 | 4%2D vs. 4%4D glycerol treatment | 0 | 0 |
| Group 4 vs. Group 7 | 4%2D vs. 1%4D glycerol treatment | 0 | 0 |
| Group 4 vs. Group 8 | 4%2D vs. 4%2D glycerol treatment | 0 | 0 |
| Group 4 vs. Group 6 | 4%2D vs. 1%2D glycerol treatment | 0 | 0 |
| Group 4 vs. Group 1 | 4%2D vs. Native | 0 | 0 |
| Group 4 vs. Group 10 | 4%2D vs. Reference | 0 | 0 |
| Group 3 vs. Group 2 | 1%4D vs. 1%2D | 0.006 | 0.284 |
| Group 3 vs. Group 5 | 1%4D vs. 4%4D | <.001 | 0.009 |
| Group 4 vs. Group 3 | 1%4D vs. 4%4D glycerol treatment | 0 | 0 |
| Group 3 vs. Group 7 | 1%4D vs. 1%4D glycerol treatment | 0 | 0 |
| Group 3 vs. Group 8 | 1%4D vs. 4%2D glycerol treatment | 0 | 0 |
| Group 3 vs. Group 6 | 1%4D vs. 1%2D glycerol treatment | 0 | 0 |
| Group 3 vs. Group 1 | 1%4D vs. Native | 0 | 0 |
| Group 3 vs. Group 10 | 1%4D vs. Reference | 0 | 0 |
| Group 2 vs. Group 5 | 1%2D vs. 4%4D | 0.323 | 1 |
| Group 2 vs. Group 9 | 1%2D vs. 4%4D glycerol treatment | 0 | 0 |
| Group 2 vs. Group 7 | 1%2D vs. 1%4D glycerol treatment | 0 | 0 |
| Group 2 vs. Group 8 | 1%2D vs. 4%2D glycerol treatment | 0 | 0 |
| Group 2 vs. Group 6 | 1%2D vs. 1%2D glycerol treatment | 0 | 0 |
| Group vs. Group 1 | 1%2D vs. Native | 0 | 0 |
| Group 2 vs. Group 10 | 1%2D vs. Reference | 0 | 0 |
| Group 5 vs. Group 9 | 4%4D vs. 4%4D glycerol treatment | 0 | 0 |
| Group 5 vs. Group 7 | 4%4D vs. 1%4D glycerol treatment | 0 | 0 |
| Group 5 vs. Group 8 | 4%4D vs. 4%2D glycerol treatment | 0 | 0 |
| Group 5 vs. Group 6 | 4%4D vs. 1%2D glycerol treatment | 0 | 0 |
| Group 5 vs. Group 1 | 4%4D vs. Native | 0 | 0 |
| Group 3 vs. Group 10 | 4%4D vs. Reference | 0 | 0 |
| Group 9 vs. Group 7 | 4%4D glycerol treatment vs. 1%4D glycerol treatment | 0 | 0 |
| Group 9 vs. Group 8 | 4%4D glycerol treatment vs. 4%2D glycerol treatment | 0 | 0 |
| Group 9 vs. Group 6 | 4%4D glycerol treatment vs. 1%2D glycerol treatment | 0 | 0 |
| Group 9 vs. Group 1 | 4%4D glycerol treatment vs. Native | 0 | 0 |
| Group 9 vs. Group 10 | 4%4D glycerol treatment vs. Reference | 0 | 0 |
| Group 7 vs. Group 8 | 1%4D glycerol treatment vs. 4%2D glycerol treatment | 0.002 | 0.016 |
| Group 7 vs. Group 6 | 1%4D glycerol treatment vs. 1%2D glycerol treatment | <.001 | 0 |
| Group 7 vs. Group 1 | 1%4D glycerol treatment vs. Native | 0 | 0 |
| Group 7 vs. Group 10 | 1%4D glycerol treatment vs. Reference | 0 | 0 |
| Group 8 vs. Group 6 | 4%2D glycerol treatment vs. 1%2D glycerol treatment | <.001 | 0.002 |
| Group 8 vs. Group 1 | 4%2D glycerol treatment vs. Native | 0 | 0 |
| Group 8 vs. Group 10 | 4%2D glycerol treatment vs. Reference | 0 | 0 |
| Group 6 vs. Group 1 | 1%2D glycerol treatment vs. Native | 0 | 0 |
| Group 6 vs. Group 10 | 1%2D glycerol treatment vs. Reference | 0 | 0 |
| Group 1 vs. Group 10 | Native vs. Reference | 0 | 0 |

**Supplemental Table 1.** A summary of statistical analyses used to estimate significant variations in optical transmittance recorded from native and decellularization samples with and without glycerol treatment. Group 1 = Native, Group 2 = 1%2D, Group 3 = 4%4D, Group 4 = 1%4D, Group 5 = 4%4D, Group 6 = 1%2D glycerol , Group 7 = 1%4D glycerol, Group 8 = 4%2D glycerol treatment, Group 9 = 4%4D glycerol treatment, and Group 10 = reference.
